# Appendicitis: A Plea for Immediate Operation

**Published:** 1914-09

**Authors:** 


**Appendicitis : a Plea for Immediate Operation.** By EDMUND OWEN, F.R.C.S., D.Sc. (Hon.). Pp. xii, 214. Bristol : John Wright & Sons Ltd. 1914. Price, 3s. 6d. net.

We welcome Mr. Edmund Owen's book on appendicitis, as it is likely to impress those who have to treat patients suffering from this disease—the general practitioner, the physician and the surgeon—with the danger which a non-operative treatment involves, and the impossibility of estimating from the symptoms and the physical examination of the patient how serious the mischief in the appendix may be, and therefore how great may be the danger of perforation or gangrene of the appendix, with serious infection of the peritoneal cavity. The author maintains that the proper treatment for all cases of appendicitis is operation as soon as the diagnosis can be made, and he shows that if this treatment is adopted statistics indicate that the mortality of the disease is considerably reduced in a large number of consecutive cases. He is quite prepared to admit that if this practice is followed a good many cases will be operated on that might have recovered without, but he considers, and we think rightly, that it is better to have an operation performed which may not really have been necessary, rather than incur the risks of an uncontrollable peritoneal infection, with the likelihood of recurrent attacks even if the patient recovers from his present one, any of which may be a source of great danger.

Mr. Owen, and indeed most surgeons, consider that after recovering from even the first attack of appendicitis, the appendix should be removed, but to this we do not subscribe. If the surgeon holds this view, why not remove it as early as possible in the first attack, though there may be more risk than in operating after the attack, when it is almost nil? The risk is very little if the operation is done quite early and the very grave risk of the continual progress of the disease avoided. Of course, if we operate quite early in the attack we have no time to watch the patient, and therefore we are more likely to err in diagnosis than if we waited a few days. We think the surgeon ought to be pretty sure of his diagnosis before he operates, without waiting for confirmation by further observation of the patient. Mr. Owen tells us that if we are suspicious only of appendicitis we must "look and see." We do not feel altogether ready to agree about this. We should certainly agree that if you can make a definite diagnosis of appendicitis it would not be wise to wait and see if any indication was forthcoming as to the exact condition of the appendix, or even whether it was seriously affected or not; but it does not seem to us that operation should be undertaken when there is only a suspicion the case may be one of appendicitis.

Indeed, Mr. Owen does not really seem quite sure about the advisability of operating in doubtful cases himself, for he says there must be an agreement of two medical men that the case is probably appendicitis before the surgeon looks to see. At any rate, we think the patient ought to be clearly informed that his medical advisers do not feel sure whether he has appendicitis or not, and they propose to "look and see." Even Mr. Owen allows that there must be "no reckless haste in advising operation." We do not think Mr. Owen sufficiently admits the difficulties that may arise in the differential diagnosis of appendicitis. For instance, he says "the surgeon who, ever on the look-out for appendicitis in his daily work, is so keen to recognise it, that he sees it in the pain and vomiting of ureteral calculus, is somewhat like a man walking up a marsh for snipe, who is so anxious to be ready for the bird that he lets off his gun even when a startled lark makes an upward dash." But the differential diagnosis between a stone in the right ureter and appendicitis may after a careful consideration be most difficult, and in several cases the appendix has been removed when a stone in the ureter caused the symptoms. Nor does Mr. Owen, we think, sufficiently admit the difficulty in the diagnosis of acute chest affections from appendicitis. He says, "Percussion and auscultation give clear evidence of the nature of the affection, but the physical signs may not be at all clear at the commencement of lobar pneumonia, and it may not always be easy to discover a pleuritic friction sound." No reference is made to the distinction to which the late Mr. Harold Barnard called attention, that "the abdominal wall becomes soft for a minute at each inspiration, which is not the case in acute peritonitis."

We think some of the most valuable information in Mr. Owen's book is that given in the chapter on "Diagnosis," with reference to the way in which the practitioner may be misled by the absence of the usual signs and symptoms, and also in the chapter on "A Fool's Paradise Stage," in which the author shows how with the subsidence of some symptoms serious mischief may yet be progressing. We are glad to see the fact that there may be no rigidity at all in acute cases is recognised, for its value as a sign, when present, may induce some to regard its absence as of distinctive negative value.

There is one detail in Mr. Owen's operative procedure we are rather surprised at. He tells us that, having opened the peritoneal cavity, he proceeds to remove the appendix, and may have to break through an abscess cavity to reach it; but we hear nothing about that packing off of the infected area from the general peritoneal cavity with gauze, before the pus is liberated, which adds so much to the safety of the operation. It may be that Mr. Owen has forgotten to mention it, and will do so in a later addition; we can hardly think he omits this precaution.

However, the value of the book lies in its lucid argument for immediate operation in appendicitis, and it will indeed do a good work if it will persuade those in charge of such cases to call in a surgeon at once, and the surgeon to operate at once if he can definitely diagnose acute appendicitis. We also sincerely hope, but hardly expect, that the book will fall into the hands of a few physicians who think they can tell what course a case of appendicitis is running by their observation of the patient. It may save them from a few of those painful surprises which the practitioner, too confident in his own power of diagnosis and prognosis with regard to this often misleading disease, will inevitably experience.

